# A Rapid Hairy Root-Based Platform for CRISPR/Cas Optimization and Guide RNA Validation in Lettuce

**DOI:** 10.3390/plants15081161

**Published:** 2026-04-09

**Authors:** Alberico Di Pinto, Valentina Forte, Chiara D’Attilia, Marco Possenti, Barbara Felici, Floriana Augelletti, Giovanna Sessa, Monica Carabelli, Giorgio Morelli, Giovanna Frugis, Fabio D’Orso

**Affiliations:** 1 National Research Council (CNR), Institute of Molecular Biology and Pathology (IBPM), 00185 Rome, Italy; alberico.dipinto@uniroma1.it (A.D.P.); giovanna.sessa@cnr.it (G.S.); monica.carabelli@cnr.it (M.C.); 2 Department of Molecular Medicine, University La Sapienza, 00161 Rome, Italy; 3 Council for Agricultural Research and Economics (CREA), Research Centre for Genomics and Bioinformatics, 00178 Rome, Italy; valentina.forte@crea.gov.it (V.F.); marco.possenti@crea.gov.it (M.P.); barbara.felici@crea.gov.it (B.F.); floriana.augelletti@gmail.com (F.A.); giorgio.morelli.crea@gmail.com (G.M.); 4 National Research Council (CNR), Institute of Agricultural Biology and Biotechnology (IBBA), 00010 Rome, Italy; chiara.dattilia@unitus.it; 5 Department of Agriculture and Forest Sciences (DAFNE), University of Tuscia, 01100 Viterbo, Italy; 6 Accademia Nazionale dei Lincei, 00165 Rome, Italy

**Keywords:** lettuce, plant development, HD-ZIP II transcription factors, hairy roots, CRISPR/Cas system

## Abstract

Cultivated lettuce (*Lactuca sativa* L.) is a major leafy crop and an emerging model for functional genomics within the Asteraceae family, supported by high-quality reference genomes and efficient transformation systems. Although CRISPR/Cas technology offers powerful opportunities for crop improvement, editing efficiency depends on optimized construct architecture and reliable guide RNA (gRNA) validation. However, a rapid platform for evaluating CRISPR reagents in lettuce is still lacking. Here, we developed an efficient hairyroot-based system to accelerate CRISPR/Cas genome editing optimization in *L. sativa*. Four *Agrobacterium rhizogenes* strains were compared for hairy root induction in two cultivars, ‘Saladin’ and ‘Osiride’, identifying strain ATCC15834 as the most effective based on transformation frequency and root production. Using this platform, we evaluated multiple CRISPR construct configurations, including alternative promoters for nuclease and gRNA expression. A plant-derived promoter combined with At-pU6-26 variant significantly improved editing efficiency. As a proof of concept, we targeted *LsHB2*, the putative ortholog of *Arabidopsis thaliana ATHB2*, a key regulator of the shade avoidance response using SpCas9, SaCas9, and LbCas12a nucleases. The system enabled rapid genotyping and quantitative indel profiling. Overall, this workflow provides a robust framework for efficient guide selection and construct optimization in lettuce genome editing.

## 1. Introduction

Cultivated lettuce (*Lactuca sativa* L.), a diploid member of the Compositae (also known as Asteraceae) family, is a leafy vegetable of considerable economic importance. It is one of the most widely grown species through indoor agriculture and vertical farming, and its breeding programs are of crucial importance for increasing yield, nutritional quality, and disease resistance. Currently, the availability of a high-quality reference genome of *L. sativa* [1,2] and many other *Lactuca* accessions [3], together with stable transformation and regeneration protocols [4,5,6], have made lettuce a model plant of the Asteraceae family suitable for genome editing approaches for functional genomics and biotechnology purposes.

CRISPR/Cas is a powerful technology that is changing the way plant scientists face applied and basic research by facilitating targeted genome engineering [7]. It is based on the action of programmable endonucleases, which cut on a specific DNA site recognized by a base pairing mechanism between a guide RNA (gRNA) assembled with the cas nuclease and the target sequence on the genome [8]. DSBs are mainly repaired by a non-homologous end joining (NHEJ) pathway; this process is error-prone and induces insertions/deletions (indels), leading to site-specific genetic perturbations [8,9]. This technology has been successfully employed to generate new plant genotypes with enhanced agronomic traits in many plant species [7,8], including lettuce [10,11].

Having a good transformation system available (to optimize the number of transformants) and using very efficient constructs (to increase the editing efficiency) is a pivotal prerequisite to fully exploit the power and effectiveness of the CRISPR/Cas system. Optimization of CRISPR constructs requires choosing the most appropriate expression system for CRISPR reagents, as well as selecting guides with high editing performance. The efficiency of the guides depends on a number of variables like nucleotide composition [12,13,14,15], GC percentage [12,16,17], and secondary structure [16,17]. To facilitate the selection of guides with high efficiency and specificity, several algorithms have been developed for SpCas9 (reviewed in [18]), Cas12a [19], and SaCas9 [20]. Unfortunately, despite their exceptional value, these computational tools have some limitations, especially when the designed guides are used in biological systems different from those used to train machine- and deep-learning-based software [21,22]. In the absence of plant-based algorithms, it is recommended to experimentally validate the CRISPR constructs before stable transformation [23].

The most used systems for guide validation include in vitro assay, agroinfiltration, protoplasts, and, more recently, hairy roots. The hairy-root-based assay offers several advantages in terms of rapidity and cost-effectiveness, allowing accurate and robust genotype analysis [23].

Several studies have shown the induction of hairy roots from different *L. sativa* cultivars [24] and other *Lactuca* species, *L. virosa* [25] and *L. indica* [26], highlighting that *A. rhizogenes* can efficiently transform lettuce explants. However, to the best of our knowledge, protocol optimization comparing the efficiency of different *A. rhizogenes* strains, as well as the use of lettuce hairy roots to validate CRISPR reagent, is still lacking.

In this work, we established an efficient protocol to induce hairy roots in *L. sativa* and optimize CRISPR plasmid features to increase editing efficiency. As a proof of concept for developmental biology studies, we targeted the lettuce gene *LsHB2*, the putative ortholog of the *Arabidopsis* HD-Zip II gene *ATHB2* that is known for its role in shade avoidance response (SAR) [27,28]. SAR is a phenomenon triggered by mutual shading provoked by the high-density cultivation widely adopted in agriculture; it occurs in numerous crop species and is considered one of the major yield-limiting factors [29,30,31,32]. Suppressing or modulating the SAR, for example, by modifying the activity of HD-Zip II genes, could be a promising strategy to optimize growth performance under crowded cultivation. *LsHB2* was selected because its predicted role in regulating growth and light responses makes it a relevant target for genome editing in lettuce.

HD-ZIP II transcription factors exhibit a conserved modular structure that confers functional specificity; in particular, they are characterized by a homeodomain (HD) responsible for sequence-specific DNA binding, immediately followed by a leucine zipper (LZ) domain that supports dimerization and stabilizes DNA interaction [33]. AtHB2 and LsHB2 display a high degree of protein sequence conservation, especially within the HD-ZIP domain, where sequence identity reaches approximately 95%. The homeodomain (HD) is a conserved 60-amino acid motif that folds into a characteristic three-helix structure that is able to interact specifically with DNA, and several aminoacidic residues within helix 3 have been shown to be essential for this activity [34]. Accordingly, we strategically targeted the *LsHB2* gene using the CRISPR/Cas system to disrupt its DNA-binding domain with the aim of generating mutants that could exhibit a reduced shade avoidance response.

We first assessed the ability of four different *A. rhizogenes* strains to induce hairy roots from cotyledon explants of two *L. sativa* varieties, ‘Saladin’ (iceberg/crisphead type) and ‘Osiride’ (romaine type). The ATCC15834 strain was selected as the most effective in terms of the number of explants producing hairy roots and the number of hairy roots per explant, and it was used for subsequent analysis to optimize the expression of nuclease and gRNAs and to test the guide’s efficiency.

Because CRISPR construct architecture is widely recognized as a key determinant of genome editing efficiency due to its direct effect on the expression levels of CRISPR reagents (nuclease and guide RNA) [35,36,37], we evaluated two distinct promoters, one viral-derived and one of plant origin, for GFP expression in lettuce hairy roots and additionally compared two U6 promoter variants to improve guide RNA expression.

The most effective promoters for both the nuclease and the guide RNA were identified and subsequently employed to evaluate CRISPR constructs harboring different nucleases, including SpCas9, SaCas9, and LbCas12a, in lettuce hairy roots, with the *LsHB2* gene serving as a developmental target.

Overall, this study provides an optimized plasmid structure to express CRISPR machinery and a valuable hairy-root-based in vivo system to finely characterize multiple guides and Cas nucleases in the leafy crop *L. sativa*.

## 2. Results

### 2.1. Selection of Efficient A. rhizogenes Strains Inducing Hairy Roots in Lettuce

Hairy root-based transformation systems are widely used as rapid platforms to assess sgRNA efficiency and genome editing activity in several plant species [38,39,40,41,42]. However, their performance is species-dependent and not directly transferable across crops, thus necessitating targeted optimization and validation for application in lettuce.

**Figure 1:**
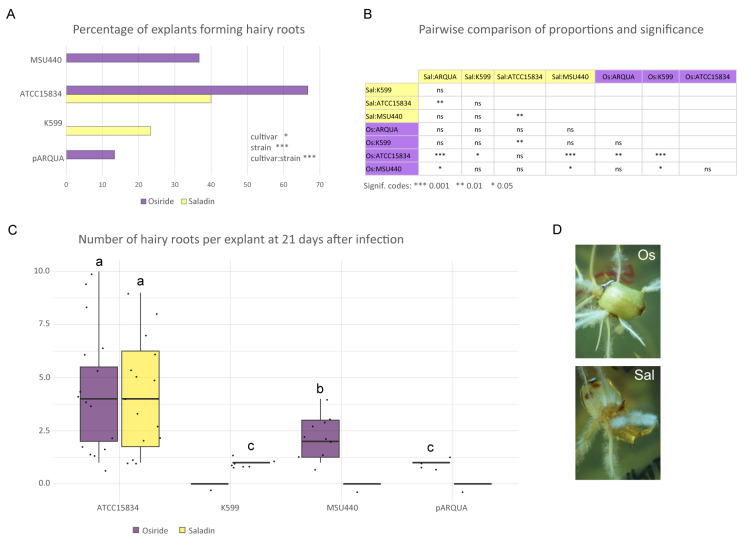
Comparative analysis of infection efficiency of different *A. rhizogenes* strains in lettuce cotyledon explants. (**A**) Infection efficiency was evaluated as the percentage of explants producing hairy roots 21 days after inoculation in the cultivars ‘Osiride’ (Os) and ‘Saladin’ (Sal). Strains exhibited variable transformation efficiencies, with strain ATCC15834 showing significantly higher infection rates compared to strains MSU440, K599, and pARQUA. The percentage was calculated based on a total of 30 explants per strain–cultivar combination. (**B**) Significant differences in the proportion of explants forming hairy roots among treatments were evaluated using the Pearson’s chi-square (χ^2^) test of independence complemented by Fisher’s exact test to ensure robustness. (**C**) Distribution of hairy root numbers per lettuce cotyledon explant following infection with different *A. rhizogenes* strains. Each point represents an individual explant; horizontal lines indicate the median. Hairy roots were counted at 21 days post-infection. Boxes represent the interquartile range, with the median indicated. Statistical significance was assessed through one-way ANOVA followed by Tukey’s HSD test; groups with different letters differ significantly (*p* < 0.05). (**D**) Representative images of cotyledons from ‘Osiride’ (Os) and ‘Saladin’ (Sal) cultivars infected with *A. rhizogenes* ATCC15834 strain.

As a first step, in this work, four different *A. rhizogenes* strains commonly used for plant transformation (ATCC15834, MSU440, K599, and pARQUA) harboring the FD165_pNos_NPTII construct (empty vector) were evaluated to determine the most efficient strain for hairy root (HR) induction in lettuce cotyledon explants. *Agrobacterium* strains were tested on two lettuce cultivars, ‘Osiride’ (romaine type) and ‘Saladin’ (iceberg/crisphead type). For each strain–cultivar combination, 30 cotyledon explants were used, and the formation of the first hairy roots was observed 7–10 days after infection. Final hairy root formation was evaluated 21 days post-infection by counting the number of hairy roots per lettuce cotyledon explant. The ATCC15834 strain could induce hairy root formation in both cultivars, although with different percentages of explants forming HR, which were close to 40% in ‘Saladin’ and around 70% in ‘Osiride’ (Figure 1A). MSU440 could induce HR only in ‘Osiride’ with a percentage of about 33, whereas K599 and pARQUA displayed low percentages of explants forming HR. These two strains were also cultivar-specific, as K599 induced HR in ‘Saladin’ explants only (around 20%), while pARQUA induced HR only in ‘Osiride’ (around 10%) (Figure 1A). Statistical analysis indicates that both the strain and the cultivar had significant effects on the response variable, and a significant strain × cultivar interaction was also observed (Figure 1A). Pairwise comparisons of proportions revealed that transformation efficiency differed significantly depending on the strain–cultivar combination (Figure 1B). In particular, ATCC15834 showed significantly higher infection efficiency compared to pARQUA and MSU440 in both cultivars. The highest response was observed in ‘Osiride’ inoculated with ATCC15834, which also differed significantly from most other combinations. In contrast, pARQUA and MSU440 generally showed low or intermediate responses, with limited significant differences between them. The strain K509 exhibited variable behavior, with moderate activity in ‘Saladin’ but no response in ‘Osiride’, contributing to some of the observed differences. Overall, these results indicate that strain ATCC15834 is the most effective for hairy root transformation, while other strains show lower or more variable efficiencies.

ATCC15834 was also the most efficient strain in terms of the number of HR induced per explant, with an average of four hairy roots in both lettuce cultivars (Figure 1C). Among the other strains, MSU440 produced an average of two HR per explant only in ‘Osiride’, while a maximum of one root was formed in explants infected with K599 and pARQUA, which displayed the lowest average root induction efficiency in lettuce explants. Analysis of hairy roots from the different strain–cultivar combinations revealed that more than 90% were positive for *NPTII* gene insertion. An example of HR genotyping is shown in Supplementary Figure S1.

Overall, the results obtained demonstrate that ATCC15834 is the most effective strain for inducing hairy roots in lettuce and that the transformation efficiency is extremely high. Although ‘Osiride’ was more receptive to transformation across a wider range of strains, both cultivars can be used effectively once the optimal strain (ATCC15834) is employed (Figure 1D).

These features make the hairy root system ideal for obtaining many independent transgenic events in a short time. For this reason, we decided to use the ATCC15834-based hairy root system for the validation of CRISPR constructs, a crucial step to verify editing efficiency before undertaking labor-intensive and time-consuming stable transformation. ‘Saladin’ was selected as the cultivar of choice for hairy root system optimization, as it is genetically closer to ‘Salinas’, which represents the reference genome for lettuce, making it ideal for genome editing studies requiring accurate sequence information.

### 2.2. Promoter Selection to Drive Efficient Gene Expression in Lettuce Hairy Roots

It is well known that the architecture of CRISPR plasmids is important, as it affects the expression of CRISPR machinery [36,37]. Different promoters driving the nuclease and gRNAs can result in markedly different editing efficiencies [35]. In lettuce, several virus- or plant-derived promoters have been used to express SpCas9 for gene editing experiments [43,44,45,46]. However, data on the expression performance of virus- and plant-derived promoters in lettuce hairy roots are currently lacking.

**Figure 2:**
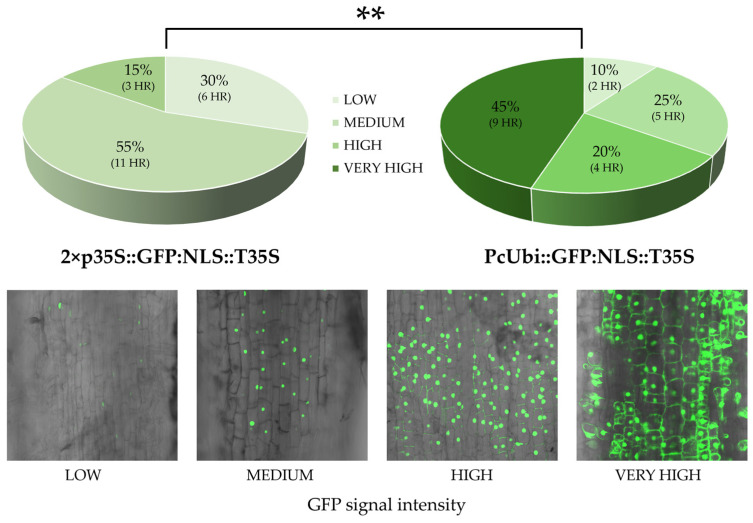
Comparative analysis of GFP expression levels in lettuce hairy roots (HR) transformed with 2×p35S::GFP:NLS::T35S and pPcUbi::GFP:NLS::T35S constructs used to evaluate the performance of the two promoters (n = 20). Hairy roots were classified by GFP signal intensity: low (slight nuclear fluorescence), medium (good nuclear fluorescence), high (strong nuclear and cytoplasmic fluorescence), and very high (extremely intense and diffuse nuclear and cytoplasmic fluorescence signal). Statistical significance was assessed using Fisher’s exact test (** *p* < 0.01).

Here, as a preliminary step prior to the validation of CRISPR constructs, we compared two promoter configurations driving the expression of a Green Fluorescent Protein (GFP) fused to a nuclear localization signal (NLS) in lettuce hairy roots. In one construct, GFP expression was driven by the cauliflower mosaic virus double 35S promoter fused to the tobacco mosaic virus omega translational enhancer (2×p35S–Ω) and in the other by the parsley ubiquitin promoter (pPcUbi) (Figure 2).

2×p35S-Ω::GFP:NLS::T35S and pPcUbi::GFP:NLS::T35S constructs were introduced into the *A. rhizogenes* strain ATCC15834 to produce transformed hairy roots, and GFP expression was visualized using confocal fluorescence microscopy. To compare the performance of the two transcriptional units, hairy roots were grouped into four expression classes based on GFP fluorescence intensity. Roots showing faint fluorescence restricted to the nucleus were classified as ‘low’, whereas those with clear and well-defined nuclear fluorescence were considered ‘medium’. ‘High’ GFP intensity corresponded to roots displaying strong fluorescence in both the nucleus and the cytoplasm. Finally, roots exhibiting extremely intense and diffuse fluorescence throughout the nucleus and the cytoplasm were categorized as ‘very high’.

While both constructs generated GFP-positive hairy roots, their expression profiles differed significantly. The pPcUbi::GFP:NLS::T35S construct produced a high proportion of events with very high (45%) or high (20%) GFP expression and only a small proportion of roots displaying medium (25%) or low (10%) fluorescence intensity. In contrast, the 2×p35S-Ω::GFP:NLS::T35S construct predominantly produced roots with medium (55%) or low (30%) expression levels; only 15% of roots displayed high fluorescence intensity, and no events reached the very high expression category.

This might be due to viral structure-driven gene silencing. Several studies have shown that in transgenic lettuce plants, transgenes driven by the CaMV 35S promoter can exhibit reduced expression [47,48,49,50,51,52,53]. Mishiba et al. [54] and Hirai et al. [51] reported that the 35S promoter can be subjected to hypermethylation at CpG and CpWpG sites in gentiana and lettuce, respectively.

Our results clearly show that the PcUbi promoter outperformed the 2×35S-Ω viral promoter, resulting in higher transgene expression levels in lettuce hairy roots, suggesting that this plant-derived promoter should be preferred when high expression levels are required. This insight may also be applicable to other *Asteraceae* species, such as sunflower (*Helianthus annuus* L.), which may exhibit a comparable regulatory effect in response to the CaMV 35S promoter [55].

Based on our evidence and data reported in the literature, we employed the PcUbi promoter to express different nucleases, an approach that has been widely used for plant genome editing across several plant species [56,57,58,59].

### 2.3. A Homologue of the Arabidopsis HD-Zip II Gene ATHB2 as a Target for Genome Editing Optimization in Lettuce

Transcription factors (TFs) act as master regulators of gene expression and can be strategically targeted by CRISPR/Cas to modify plant phenotypes via loss-of-function mutations or in-frame deletions, remodulating the transcriptional regulatory networks underlying complex agronomic traits.

For genome editing optimization in lettuce, we selected *LsHB2*, a gene encoding a transcription factor of the HD-ZIP II family, which is putatively involved in the shade avoidance response.

We strategically targeted the HD-coding region of the *LsHB2* gene using the CRISPR/Cas system to disrupt its DNA-binding domain, with the aim of generating mutants that could exhibit a reduced shade avoidance response.

**Figure 3:**
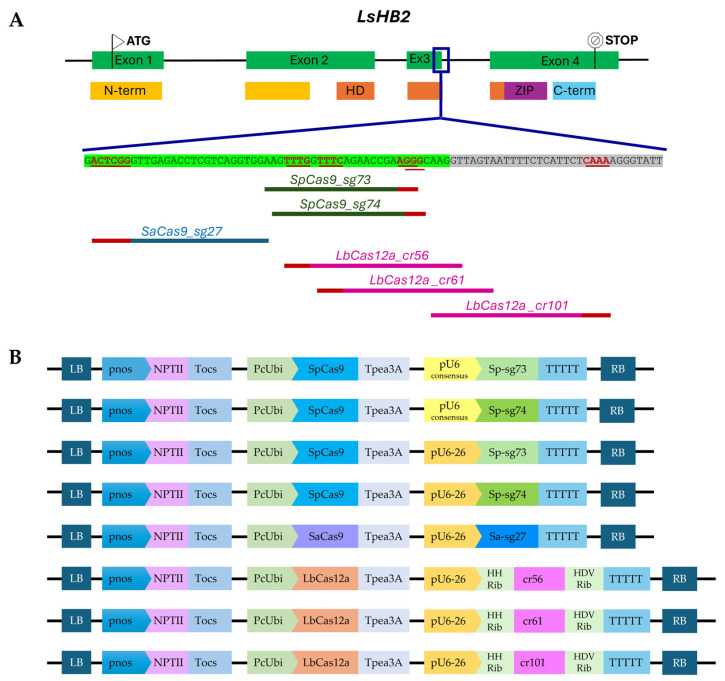
(**A**) Schematic representation of genomic organization of the *LsHB2* gene (exons are depicted as green boxes and introns as lines), with the corresponding protein domains shown below: the N-terminal domain is shown in yellow, the homeodomain (HD) in orange, the leucine zipper (ZIP) in purple, and the C-terminal domain in light blue. All guides used in this work to target the *LsHB2* gene are shown. PAM sequences are shown in red, while target sequences are shown in green, blue, or pink for SpCas9, SaCas9, and LbCas12a, respectively. (**B**) T-DNA structure of the CRISPR vectors carrying different nucleases and guide RNAs.

Guide RNA design was carried out with the CRISPOR web tool [60] by using the sequence corresponding to exon 3 (coding for part of the DNA binding domain) and the adjacent portion of intron 3 of *LsHB2* gene as input. Analyses were performed for different nucleases, including not only the most commonly used nuclease SpCas9 from *Streptococcus pyogenes* but also SaCas9 from *Staphylococcus aureus* [61] and LbCas12a from *Lachnospiraceae bacterium ND2006* [62], which are also commonly employed in plants [63,64,65]. Candidate gRNAs were manually selected based on their physical proximity to the DNA binding domain coding region and subsequently evaluated in silico for predicted editing efficiency and specificity (Table 1 and Table S8); a total of six guides were designed. For SpCas9, two candidate gRNAs with canonical NGG PAM sequences were identified within the region of interest (Sp-sg73 and Sp-sg74); these guides differ by a single nucleotide and overlap for most of their length. For SaCas9, the gRNA Sa-sg27 was identified as a suitable candidate. And, finally, for LbCas12a, multiple candidate gRNAs were identified in the region of interest, including cr56, cr61, and cr101, all displaying acceptable features (Figure 3A).

CRISPOR integrates algorithms for multiple score prediction for guide RNA efficiency, such as Fusi/Doench/Azimuth [66], CRISPRscan [14], Najm [20], and DeepCpf1 [19], which estimate the expected on-target activity of SpCas9, SaCas9, and Cpf1 gRNAs, respectively. It also incorporates the ‘Out-of-frame score’ [67], which predicts the likelihood of out-of-frame deletions induced by each sgRNA, a parameter relevant when gene knockouts are desired. All of these values for each guide are reported in Table 1.

### 2.4. Comparative Evaluation of Short Synthetic and Native U6 Promoters for Guides’ Expression Optimization

**Figure 4:**
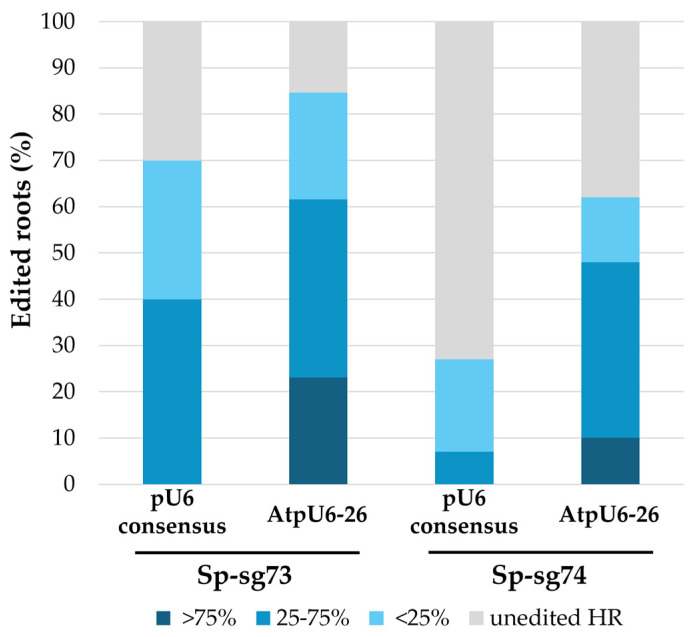
Comparison of the pU6 consensus and AtpU6-26 promoter activity based on an evaluation of the gene editing efficiency of two sgRNAs, Sp-sg73 and Sp-sg74, using the CRISPR/Cas9 system. Each bar represents the percentage of unedited (gray segment) and edited hairy roots (blue segment). Edited roots were further classified into three categories based on the contributions of each mutated allele in each root and are represented by a blue color scale; dark, medium, and light blue represent a contribution of >75%, 25–75%, and <25%, respectively.

To facilitate the cloning procedure, particularly when multiple guide cassettes need to be assembled, and to ensure plasmid stability by avoiding long repetitive sequences that could increase the risk of recombination events, the use of short promoters is advantageous. However, during the selection of an appropriate promoter, transcription efficiency should be carefully considered to maximize sgRNA expression and, consequently, CRISPR efficiency. Multiple studies have demonstrated that sgRNA expression levels are a key determinant of CRISPR-mediated editing efficiency, with higher sgRNA abundance or stronger promoters correlating with increased Cas9 activity [36,68,69]. In the pioneering work of genome editing in plants, Nekrasov and colleagues [70] developed a 75 bp long synthetic U6 promoter representing a consensus sequence of 3 *Arabidopsis thaliana* U6 promoters that has been successfully used to express sgRNAs in several plant species [22,23,70,71,72,73]. With regard to its effectiveness, to the best of our knowledge, a direct comparison between this short pU6 consensus and the more conventional and widely used pU6-26 from *A. thaliana* (AtpU6-26) is lacking. In this work, to optimize the expression of the guides in lettuce hairy roots, we compared these two U6 versions of promoters targeting two SpCas9 sites in the *LsHB2* gene using Sp-sg73 and Sp-sg74 guides. For each sgRNA, two constructs were generated that differed only in the promoter driving sgRNA expression, AtpU6-26 or pU6 consensus (Figure 3B), and these constructs were used to produce transformed hairy roots. To evaluate the efficiency of the two promoters driving sgRNA expression, editing outcomes were assessed through Sanger sequencing of PCR amplicons spanning the target region. Sanger electropherograms from transformed hairy roots were analyzed using ICE software (Synthego; https://ice.synthego.com, accessed on 20 March 2025) to quantify indel formation at the target site and ensure comprehensive detection of editing events in a complex editing profile [74]. Promoter efficiency was calculated by determining the proportion of roots containing at least one edited allele relative to unedited roots. In addition, for each edited root containing one or more editing events, we reported the ICE% score (indel frequency) ranging from 1% to 99%; accordingly, edited roots were classified into three categories based on the ICE% score in each root (>75%, 25–75%, and <25%), as shown in Figure 4.

Firstly, although the two guides were almost identical (shifted by 1 nt), they exhibited different editing efficiencies regardless of the promoter used. Sp-sg73 not only generated a higher number of edited roots compared to Sp-sg74 (70% vs. 27% for pU6 consensus and 84% vs. 62% for AtpU6-26) but also resulted in a greater proportion of hairy roots with high indel frequencies (ICE score > 75%). Promoter comparison indicated that the AtpU6-26 consistently performed better than pU6 consensus with both guides, resulting in a higher number of edited roots (84% vs. 70% for sgRNA73 and 62% vs. 27% for sgRNA74) and increased editing frequency (Figure 4). By using AtpU6-26, we observed a reduction in the proportion of roots with an ICE score < 25% and an increase in roots with an ICE score > 25% (including both ICE score = 25–75% and ICE score > 75%). It is worth noting that for both guides, roots transformed with pU6 consensus showed no events with ICE score > 75%.

Although short promoters can facilitate the construction and maintenance of multiplex CRISPR plasmids and have been shown to be effective in some plant species [22,23,70,71,72,73,75], our data indicate that in lettuce hairy roots, the synthetic pU6 consensus developed by Nekrasov et al. [70] underperformed AtpU6-26. Consequently, to achieve higher editing efficiency, AtpU6-26 was chosen. This allowed us to assess more in depth the editing performance of CRISPR constructs based on SpCas9, as well as with other nucleases, such as SaCas9 and LbCas12a.

### 2.5. Validation of CRISPR Constructs in Lettuce Hairy Roots and Comparison Between Computational Prediction and in Planta sgRNA Efficiency

By integrating all of the information obtained in the previous sections, including the conditions required to achieve high-efficiency generation of transformed lettuce hairy roots and the optimization of nuclease and guide RNA expression, we decided to further exploit this method to validate CRISPR constructs and to evaluate in planta the editing efficiency on the *LsHB2* gene.

For this purpose, besides the two SpCas9-based constructs, four additional CRISPR/Cas constructs were generated using the same expression-optimized architecture, with the PcUbi promoter driving the expression of SaCas9 or LbCas12a and the AtU6-26 promoter driving guide RNA expression (Figure 3B).

To evaluate the in planta genome editing efficiency of the guide RNAs designed on the *LsHB2* gene, the target site was amplified and sequenced from the genome of each hairy root. Sanger electropherograms were analyzed through regression analysis using ICE software, which allows not only the quantification of the number of mutated roots but also the indel frequency in each root (expressed as the ICE% score). In addition, the software predicts the number and type of mutations and their relative frequencies, enabling the assessment of whether the mutation pattern is homogeneous within each root or whether mosaicism is present, as well as the estimation of the frequency of mutations resulting in a knockout effect (KO score). For each construct, at least 10 hairy roots were analyzed for the presence of indels.

**Figure 5:**
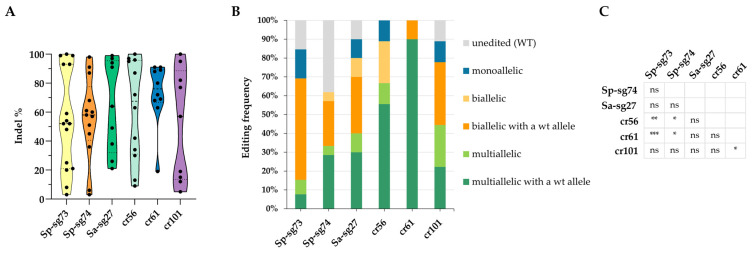
Editing frequency is guide-dependent. (**A**) Violin plots illustrate the distribution of insertion and deletion (indel) proportions generated by six different constructs in lettuce hairy roots. Each violin represents the density and range of indel events detected for a given construct. The width of the plots reflects the relative frequency of events. The data reveal distinct indel distribution patterns among constructs, highlighting variability in editing outcomes and nuclease efficiency, although no statistically significant differences were observed using one-way ANOVA (Table S11). (**B**) The graphic displays the editing frequencies obtained in hairy roots using different combinations of guides and nucleases, indicated on the *x*-axis. Each stacked bar represents the percentage distribution of the various genotypic classes detected through ICE analysis: unedited, monoallelic, biallelic, and multiallelic events with or without residual wild-type alleles. (**C**) Statistical significance of differences in the proportion of genotypic classes among different guides were assessed using Fisher’s exact test (* *p* < 0.05, ** *p* < 0.01, and *** *p* < 0.001; Table S12).

All designed sgRNAs were effective, although they displayed different mutagenesis frequencies. Distinct indel distribution patterns among constructs are shown in Figure 5A. Albeit not statistically significant (Table S11), these differences are likely attributable to the specific sequence features of each sgRNA (including their intrinsic nucleotide composition and local secondary structure), as well as the particular nuclease employed, which can influence cleavage efficiency and repair outcomes. Editing efficiency, calculated by the number of edited hairy roots out of the total hairy roots, ranged from 62% for Sp-sg74 to 100% for cr56 and cr61 (Figure 5B).

Analyses of edited hairy roots revealed different proportions of mutation types associated with the different guides and nucleases, as well as variable frequencies of unedited alleles. Therefore, hairy roots were classified into four categories according to their mutation profiles: unedited (wild type); monoallelic mutants (only one edited allele was detected, which may reflect either a homozygous mutation or the presence of additional mutations that were not detected, for example, due to impaired PCR amplification); biallelic mutants (two alleles carrying different sequences, either two distinct edited alleles or one edited and one wild-type allele); and mosaic mutants (harboring multiple distinct edited alleles with or without a wild-type allele) (Figure 5B). The Sp-sg73 guide showed a strong predominance of biallelic events with a wild-type allele and a minor fraction of monoallelic, multiallelic, and non-edited fractions, while Sp-sg74 balanced the distribution of unedited, biallelic, and multiallelic fractions. For two of the LbCas12a guides (cr56 and cr61), all hairy roots were edited, and multiallelic edits dominate the outcome distribution, resulting in statistically significant differences compared to the two SpCas9 guides (Figure 5C). On the contrary, the cr101 guide showed statistically significant differences only when compared to the cr61 guide. Cr101 and Sa-sg27 guides showed a similar and balanced profile, combining substantial multiallelic and biallelic fractions with smaller contributions from other categories. The proportions differ markedly from one guide to another, indicating guide-dependent variation in editing outcomes.

The in planta editing outcomes observed in hairy roots transformed with different CRISPR/Cas systems (SpCas9, SaCas9, and LbCas12a) were compared to computationally predicted sgRNA efficiencies to evaluate the reliability of the in silico prediction tool. Table 1 summarizes in vivo editing performance as quantified by ICE analysis, taking into consideration the percentage of edited roots, the indel frequency, and, for guides whose mutations affected specifically the exon sequence (Sp-sg73, Sp-sg74, and Sa-sg27), the KO score.

Comparison of computational predictions with the in planta outcomes revealed limited quantitative concordance, highlighting the limitation of the predictive power of current scoring algorithms. For SpCas9, editing frequencies inferred from Fusi/Doench/Azimuth and CRISPRscan scores did not reliably predict in vivo performance; in fact, although Sp-sg73 and Sp-sg74 received similar moderate predicted on-target activity scores, Sp-sg73 exhibited markedly higher editing efficiency in planta. Similarly, predicted efficiencies for LbCas12a, although more variable (ranging from 30.8 to 78.7), consistently underestimated the in planta editing outcomes, as all LbCas12a guides produced high frequencies of edited roots (89–100%). In contrast, the SaCas9 guide sg27 showed strong in vivo editing (up to 90% edited roots), consistent with its high predicted activity score, indicating that prediction accuracy may be guide- and nuclease-dependent. Predictions of frameshift frequencies based on the ‘Out-of-frame’ model tended to underestimate the knockout efficiencies observed in planta, as evidenced by the normalized KO scores, which consistently displayed higher values than the corresponding Out-of-frame scores.

Overall, these results suggest that although computational predictions can offer useful guidance, they do not always quantitatively reflect in planta editing outcomes. The predictive power of current computational scoring tools remains limited and may vary depending on the nuclease employed and the biological context. This is likely because CRISPOR models are primarily trained on mutational events observed in humans and other animal species rather than in plant systems.

This is in line with the findings of Naim et al. [21], who evaluated the predictive performance of several online tools and found no statistical correlation between software rankings and in vivo effectiveness measured in various plant species. On the contrary, more recently, Gong et al. [76] observed a significant association between the gRNA prediction scores generated by computational tools and the frequency of CRISPR-induced mutations in plant systems. Unfortunately, in both cases, a limited number of guides were analyzed, and, at present, the reliability of these computational tools remains controversial. In the absence of plant-trained algorithms, experimental in vivo validation is necessary.

Based on the results presented in this study, the hairy root system represents a valuable approach for validating sgRNA efficiency in vivo for genome editing in lettuce.

Because the choice of guide RNA for genome editing depends on specific experimental goals, the availability of an in vivo method that enables the evaluation of the type of mutations that occur under experimental conditions represents an effective approach.

**Figure 6:**
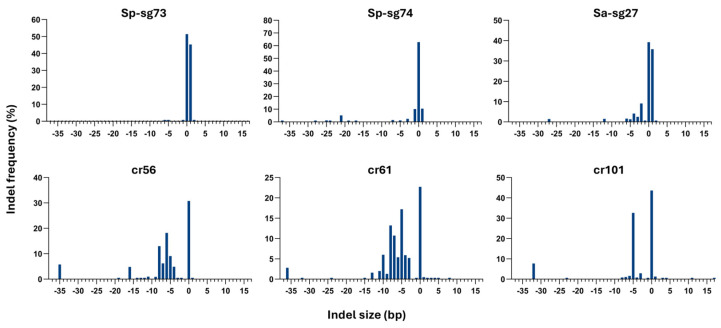
Indel profiles and corresponding frequencies in hairy roots edited with different gRNAs. Negative indel size represent deletions, positive values indicate insertions, and zero corresponds to the wild-type allele.

Coupling the hairy root transformation assay with ICE analysis enabled accurate determination of the indel profiles generated by the different gRNAs (Figure 6 and Table S1). The number of indels with an occurrence frequency higher than 5% varied for each sgRNA. The variability in indel profiles observed across the different sgRNAs indicates that editing outcomes are strongly influenced by target context; notably, SpCas9 produced a narrower indel spectrum (in most cases, only one indel) than SaCas9 and LbCas12a, suggesting higher cutting precision and more predictable DNA repair pathways (Table S1).

In general, we observed more deletions than insertions. The largest deletion observed was 37 bp long, while the largest insertion observed was 1 bp. Because ICE operates within a limited analysis window around the CRISPR cut site, typically a confidence region of approximately 30–40 bp, only indels occurring close to the cleavage position can be reliably detected. Consequently, long indels or complex rearrangements may have fallen outside of the analyzable window, leading to low R2 values (<75%) or complete failure of detection, and therefore were not included in the analysis.

**Figure 7:**
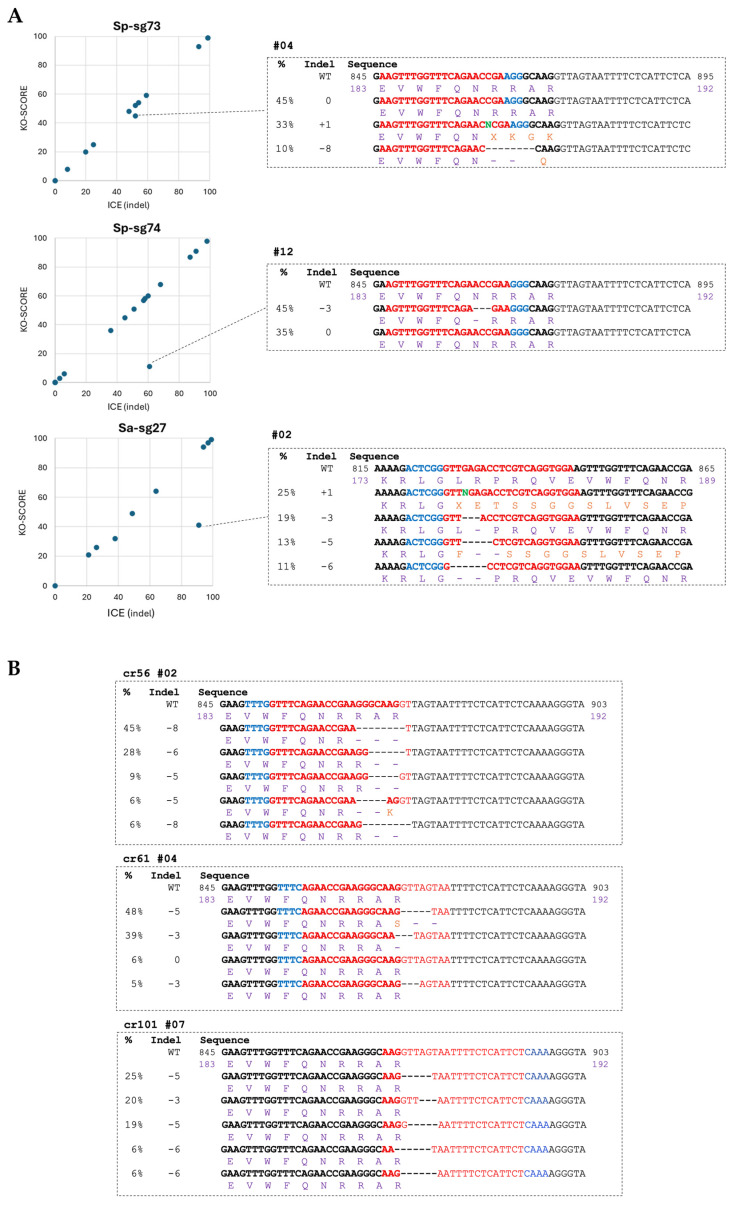
Identification and characterization of mutations induced by different guides in lettuce hairy roots. (**A**) On the left, ICE and KO score values for each individual hairy root obtained with SpCas9 and SaCas9 are plotted. On the right, a sequence alignment table displays the indel mutations induced by SpCas9 and SaCas9 guides identified in a single hairy root (frequency percentages of each indel and type of indel and its corresponding nucleotide sequence). (**B**) Sequence alignments display the indel mutations induced by Cas12a guides identified in a single hairy root. The alignments highlight the diversity and frequency of CRISPR-induced mutations within the target region. The WT sequence of the target sequence of the *LsHB2* gene is shown at the top as a reference (the exon region is indicated in bold). Red letters indicate target sequences of the guide; blue letters refer to PAM sequences; green letters indicate inserted nucleotides. Below each nucleotide sequence, the corresponding amino acid sequence is shown; the wt amino acid sequence is indicated in purple, whereas the mutated amino acids are highlighted in orange. % indicates the relative contribution of each indel. Indel indicates targeted sequence changes: ‘0’ for no change, ‘−’ for deletions, ‘+’ for insertions.

In addition, based on the type of mutation, the ICE tool provides the KO score (Figure 7A and Table 1), which estimates the percentage of the edited allele population with knockout mutations, namely, insertions or deletions not in multiples of 3 bp and large deletions (≥21 bp).

Sp-sg73 mainly induced 1 bp insertion with nearly perfect alignment of the KO score and the ICE score for almost all root samples (Figure 7A), resulting predominantly in a knockout effect on the *LsHB2* gene. On the contrary, Sp-sg74 and Sa-sg27 induced both small insertions and deletions, some of which were in-frame. Particularly interesting are the root samples Sp-sg74#12 and Sa-sg27#02 with relevant deviations of the KO score from the ICE score. The Sp-sg74#12 root showed that 45% of the detected alleles mutated with a 3 bp deletion resulting in a deletion of N188, while Sa-sg27#02 displayed several in-frame deletions with different frequencies (Table S1). Among the most representative (frequency > 10%), we found an allele with 3 bp deletion (19%) leading to the elimination of the amino acid R178 and an allele with 6 bp deletion (11%) resulting in the removal of the amino acids 177-LR-178. All of these in-frame mutations occur within the DNA-binding domain region and could therefore impair or reduce the DNA-binding capacity of the HD-ZIP transcription factor, thus rendering the dimeric protein transcriptionally inactive. Because the crRNA target regions are in the exon–intron junction, the interpretation of the possible effect of mutagenesis in those regions is more complex. Indeed, depending on the size of the deletion, it may affect the coding sequence of exon 3 or disrupt the splice site in the adjacent intronic region. Mutations at the 5′ splice site consensus sequence [77] may lead to exon skipping, intron retention, or the activation of cryptic splice sites, in most cases resulting in the synthesis of aberrant RNAs.

All crRNAs, consistent with the higher level of genetic mosaicism (Figure 5), exhibited considerable heterogeneity in indel sizes (Figure 6), producing a broad spectrum of deletions that affected both exon 3 and intron 3 (Figure 7B).

In Figure 7B, we report several examples of the effect of mutations induced by cr56, cr61, and cr101. We identified multiple alleles in which deletions removed the 5′ splice site donor GT (root samples cr56#02, cr61#04, and cr101#07). In other alleles, deletions affected only exonic nucleotides, resulting either in a knockout effect or the deletion of few amino acids. Finally, we observed alleles with small deletions that did not affect the coding sequence or the 5′ GT splice site but altered the consensus sequence downstream GT site. In this case, the effect of the mutation is less predictable and should be assessed by analyzing the transcript.

Overall, sgRNAs for SpCas9 and SaCas9 nucleases effectively induced mutations in the *LsHB2* coding sequence, mainly leading to its complete loss of function. However, at low frequency, the Sp-sg74 and Sa-sg27 guides generated in-frame mutations that alter only a limited number of amino acids, resulting in proteins with partial activity. Cas12a constructs generated a wide range of mutations, primarily deletions of various size affecting splicing, without excluding the potential disruption of gene function by inducing knockout mutations in the coding sequence.

Altogether, the results of this study enable us to have several tools available for different purposes.

## 3. Discussion

This study establishes a rapid and efficient protocol for inducing hairy roots in *Lactuca sativa*, an economically important leafy crop, providing a platform for the fast evaluation of multiple sgRNAs and promoters prior to whole-plant transformation and optimizing key CRISPR plasmid features to enhance genome editing efficiency. Although hairy root-based CRISPR validation systems have been reported in several plant species, an efficient and systematically optimized platform for evaluating CRISPR reagents in *L. sativa* has been lacking. Here, we establish and optimize such a platform, enabling rapid and reliable assessment of genome editing tools in this species. Compared to previously reported hairyroot-based CRISPR validation systems (reviewed in 2022 [78]), our platform introduces several key improvements. Kiryushkin et al. [78] note that while over 100 plant species are known to form hairy roots, many still lack optimized transformation protocols, and selecting a highly virulent strain is a critical precondition for success. To address this point, we systematically evaluated multiple *Agrobacterium rhizogenes* strains and identified ATCC15834 as providing superior transformation efficiency and faster root induction, resulting in a more robust and reproducible workflow.

Second, we provided data on promoter performance specifically in lettuce hairy roots, demonstrating that the plant-derived PcUbi promoter significantly outperforms the viral-derived 2×p35S–Ω promoter. Starting from this, we optimized the genetic architecture of the editing constructs by incorporating the plant-derived PcUbi promoter and the AtU6-26 variant, which significantly enhance nuclease and guide RNA expression in lettuce. In their review, Kiryushkin et al. [78] mention that whether using species-specific or synthetic snoRNA (U6/U3) promoters affects editing efficiency remains a point of controversy in the field. We resolved this for lettuce by conducting a direct comparison between a short synthetic pU6 consensus and the native AtpU6-26 promoter. Our results showed that the native AtpU6-26 consistently yielded higher editing efficiency and more frequent high-indel events in lettuce hairy roots than the synthetic version. 

Third, unlike earlier systems that typically assess a single nuclease (reviewed in Kiryushkin et al. [78]), our platform enables rapid and parallel validation of multiple CRISPR systems (SpCas9, SaCas9, and LbCas12a), allowing for direct comparison of their in vivo performance. This allows for rapid validation of guides before committing to the ‘labor-intensive and time-consuming’ process of stable whole-plant transformation. Further platform robustness is obtained by coupling the hairy root assay with ICE (Inference of CRISPR Edits) analysis, which allows for (i) quantitative indel profiling and the determination of ‘KO scores’ (predicting functional knockouts) and (ii) detailed assessment of mosaicism and mutation types (homozygous, biallelic, or multiallelic) within the roots. Our results highlight that empirical in vivo testing provides a more accurate assessment of editing efficiency and mutation outcomes than computational predictions. Finally, by targeting the developmental gene *LsHB2*, we demonstrate that our system enables the generation of a broad spectrum of mutations, including gene knockouts, in-frame deletions, and splice site disruptions. Collectively, these advances establish a faster, more efficient, and more informative platform for CRISPR validation in lettuce.

Regarding lettuce genotypes, differences in transformation efficiency were observed between the two lettuce cultivars ‘Osiride’ (romaine type) and ‘Saladin’ (iceberg/crisphead type), particularly in the percentage of explants forming hairy roots. ‘Osiride’ exhibited a higher transformation frequency than ‘Saladin’. The cultivars also responded differently to bacterial strains: MSU440 and pARQUA induced hairy roots only in ‘Osiride’, whereas K599 was the only additional strain capable of inducing roots in ‘Saladin’ and failed to do so in ‘Osiride’. However, despite differences in transformation frequency, the intensity of root production was similar once transformation occurred. Overall, while ‘Osiride’ is more receptive to transformation across a broader range of strains, both cultivars can be used effectively when the optimal strain (ATCC15834) is employed. ‘Saladin’ was selected for hairy root system optimization because it is genetically closer to ‘Salinas’, the lettuce reference genome, making it suitable for genome editing studies requiring accurate sequence information.

## 4. Materials and Methods

### 4.1. Plant Material

Lettuce seeds were purchased from https://www.bloomling.it/ (accessed on 20 March 2025) (cv. ‘Saladin’) or obtained from Enza Zaden Italia (cv. ‘Osiride’). For in vitro cultivation, seeds of both cultivars were immersed in 70% (*v*/*v*) ethanol for 2 min in sterile tubes with gentle mixing. The ethanol solution was then promptly removed and replaced with 5–10 mL of a 10% (*v*/*v*) sodium hypochlorite (NaClO) solution supplemented with 0.01% (*v*/*v*) Tween 20. Seeds were sterilized for 10 min while agitated at 70–100 rpm on an orbital shaker. Following sterilization, the NaClO solution was discarded, and seeds were rinsed 4–5 times with sterile distilled water, each wash lasting 3–4 min, under continuous agitation at 70–100 rpm. For germination, lettuce seeds were placed in Petri dishes or glass jars containing half-strength Murashige and Skoog medium (MS ½) supplemented with 1% (*w*/*v*) sucrose, solidified with 7 g L^−1^ agar (Micro Agar, Duchefa, Haarlem, The Netherlands) and adjusted to pH 5.8 prior to autoclaving. After autoclave sterilization, the medium was supplemented with filter-sterilized thiamine (1 mg L^−1^), pyridoxine (1 mg L^−1^), and nicotinic acid (1 mg L^−1^). Lettuce seedlings were grown under a 16 h light/8 h dark photoperiod at 22–25 °C until full cotyledon expansion, which typically occurred within 7–10 days. Cotyledons were then excised and used for *A. rhizogenes*-mediated transformation.

For the comparative analysis of *A. rhizogenes* strains, a total of 30 cotyledon explants were used, distributed across three biological replicates for each strain–cultivar combination. Formation of the first hairy roots was observed 7–10 days post-infection, and final hairy root (HR) formation was evaluated 21 days post-infection as the percentage of explants producing hairy roots.

### 4.2. Selection of Target Gene and Guide Design

In this study, as a proof of concept, we used the *LsHB2* gene (Lsat_v1_gn_6_93540), a member of the HD-ZIP II family putatively involved in the shade avoidance response, as a target for the CRISPR machinery. The gene was identified using a homology-driven approach: the Plant Transcription Factor Database (PlantTFDB) was queried with the AtHB2 amino acid sequence as input. Among the retrieved candidates, LsHB2 showed high sequence similarity and was therefore considered the putative ortholog of AtHB2 in lettuce.

Guide RNA design was carried out with CRISPOR (http://crispor.tefor.net; accessed on 20 March 2025). The reference genome used for off-target prediction was *Lactuca sativa* (garden lettuce), assembly NCBI GCA_002870075.2 (Lsat_Salinas_v7). Analyses were performed for the nucleases SpCas9, SaCas9, and LbCas12a. Candidate gRNAs were manually selected based on their physical proximity to the DNA binding domain coding region and subsequently evaluated in silico for predicted efficiency.

### 4.3. Cloning Procedure

All constructs in this study were built by using Golden-Gate-based Modular Cloning (MoClo) system [79]. Details on cloning reactions are reported in Supplementary File S1 and Table S6.

### 4.4. Hairy Root Protocol

The following conditions were applied to all hairy root experiments: comparison of several *A. rhizogenes* strains (ArQUA, K599, ATCC15834, MSU440), selection of promoters for nuclease expression, and validation of CRISPR construct. *A. rhizogenes* strains were freshly grown in 20 mL of TY medium (5 g L^−1^ Tryptone, 3 g L^−1^ yeast extract, 1.325 g L^−1^ CaCl_2_·6H_2_O) supplemented with the appropriate selection of antibiotics and incubated at 28 °C with shaking for 1 day until an OD_600_ of 0.8 was reached. Subsequently, bacterial cells were harvested through centrifugation at 3000 rpm for 20 min and resuspended in 40 mL of liquid MS medium (1× MS salts, 30 g L^−1^ sucrose, 0.5 g L^−1^ MES; pH 5.7) to obtain a final OD_600_ of approximately 0.4. At this stage, the *A. rhizogenes* cultures were ready for cotyledon transformation, and 7-day-old lettuce seedlings were used for explant preparation. Fully expanded cotyledons were excised by cutting at the base of the petiole, immediately immersed in MS medium (1× MS salts, 30 g L^−1^ sucrose, 0.5 g L^−1^ MES; pH 5.7), and trimmed at both apical and basal ends using a sterile scalpel. When cotyledons were particularly elongated, they were further divided into two segments, ensuring that final explants were not shorter than 0.4–0.5 cm. After excision, cotyledon explants were gently transferred into a new Petri dish containing the *A. rhizogenes* culture suspensions and incubated for 20 min while gently agitating the plates to ensure uniform contact between explants and bacteria. Following incubation, explants were recovered using sterile forceps and placed onto three overlapping sterile Whatman filter paper discs in a Petri dish to remove excess bacterial suspension. Explants were then individually transferred to new Petri dishes containing co-cultivation medium (1× MS salts, 30 g L^−1^ sucrose, 0.5 g L^−1^ MES, 8 g L^−1^ Plant Agar; pH 5.7; after autoclaving, they were supplemented with thiamine 1 mg L^−1^, pyridoxine 0.5 mg L^−1^, and nicotinic acid 0.5 mg L^−1^), positioning them with the abaxial side facing upward (adaxial side in contact with the medium). Fifteen to twenty explants were placed per plate, with a total of three plates for each construct. Plates were sealed with Micropore tape and incubated in the dark at 25 °C for 3 days. After 3 days of co-cultivation, explants were transferred to Petri dishes containing selection/induction medium (identical to the co-cultivation medium and supplemented with 200 mg L^−1^ cefotaxime sodium and 100 mg L^−1^ kanamycin to select hairy roots transformed with GFP or CRISPR constructs), maintaining the same orientation (abaxial side facing upward) and ensuring that the cut surfaces were in close contact with the culture medium without damaging the leaf lamina. For each construct, approximately 40 explants were transferred to a selection/induction medium, with 10 to 12 explants per plate and a total of four plates. Plates were sealed with Micropore tape and incubated in the dark at 25 °C for 2–4 weeks. At the end of this period, hairy roots were induced and sufficiently developed to allow for excision and subsequent genotyping or confocal visualization.

### 4.5. Hairy Roots Genotyping

Hairy root genotyping was performed by using Phire Plant Direct PCR Kit (Thermo Fisher Scientific Baltics UAB, Vilnius, Lithuania). Briefly, small pieces of hairy roots (approximately 1 cm) were homogenized in 30 µL of dilution buffer together with glass beads and using TissueLyser II (Qiagen) at the max frequency for 3 min. The homogenate was centrifuged at 13,000 rpm for 5 min, and 0.5 µL of supernatant were used directly as a template for PCR amplification of the target genes. PCR products were visualized on agarose gel electrophoresis and, in the case of *LsHB2*, the amplicons were sequenced by an external service to assess editing efficiency. Primers used for amplification are reported in Table S7.

### 4.6. GFP Visualization

Hairy roots generated from transformed cotyledonary explants were excised and mounted on slides in distilled water. Differential interference contrast (DIC) and confocal microscopy were used to analyze the expression pattern and intensity of GFP constructs. The analyses were performed on an inverted Z.1 microscope (Zeiss, Oberkochen, Germany) equipped with a Zeiss LSM700 spectral confocal laser scanning unit and equipped with a differential interference contrast microscopy (DIC) tool. Samples were excited with a 488 nm, 10 mW solid laser and an emission wavelength of 520 nm. In order to compare the GFP signal intensity between different roots and between the two constructs, the acquisition system was calibrated on a root transformed with the construct 2×p35S-Ω::GFP:NLS::T35S so that the GFP-expressing nuclei were appreciable and the intensity was not saturated. All 2×p35S-Ω::GFP:NLS::T35S and pPcUbi::GFP:NLS::T35S hairy roots were analyzed using these settings.

### 4.7. Bioinformatic Tools for Electropherogram Deconvolution

The computational tool ICE (Inference of CRISPR Edits) was employed to estimate in planta editing efficiency [74] (accessed on 20 March 2025). It was designed to infer CRISPR-induced indels from Sanger sequencing data by deconvoluting mixed sequencing traces obtained from heterogeneously edited cell populations.

Electropherogram files (.ab1 format) generated through Sanger sequencing of PCR amplicons of a control (unedited) sample and CRISPR-edited samples were provided to ICE software together with the target site sequence and the type of nuclease to predict the expected cleavage site and to define the alignment window and the region of interest for indel inference.

The key output parameters provided by ICE are the ICE score (overall editing efficiency, calculated as the percentage of mutated alleles relative to the total allelic population), KO score (proportion of alleles carrying frameshift mutations or large deletions likely to cause functional knockout), Indel spectrum (quantitative distribution of predicted indel sizes and their relative frequencies), and R2 (also referred to as Goodness-of-Fit, representing the confidence in the deconvolution).

Only ICE analyses with an R^2^ > 0.75 were considered reliable. INDEL and knockout (KO) scores generated by ICE were calculated for individual hairy roots and reported as a percentage. To summarize the overall editing outcomes for each guide RNA, all indel events detected in hairy roots obtained with the same guide (minimum n = 10) were analyzed, and the frequency of each indel was calculated using Excel across all analyzed roots for that guide.

### 4.8. Statistical Analyses

Statistical analyses for hairy root transformation efficiency were conducted in R 4.3.3 (R Core Team, 2021). For the analysis in Figure 1A,B, differences in the proportion of explants forming hairy roots among treatments were evaluated using Pearson’s chi-square (χ^2^) test of independence complemented by Fisher’s exact test. A contingency table was constructed using the number of explants forming hairy roots for each combination of cultivar and *A. rhizogenes* strain (Table S9). Expected frequencies were calculated under the assumption of independence between factors. The χ^2^ statistic was used to test for significant associations between cultivar and strain effects on hairy root induction.

For the analysis in Figure 1C and Figure 5A, statistical significance was assessed using one-way ANOVA followed by Tukey’s HSD test; differences were considered statistically significant at *p* ≤ 0.05. Box plots (Figure 1B) was obtained using the R ggplot2 package [80] (https://ggplot2.tidyverse.org, accessed on 20 March 2025). For the statistical analysis in Figure 2 and Figure 5B, contingency tables were constructed and statistical significance was assessed using Fisher’s exact test (*p < 0.05, ** *p* < 0.01 and *** *p* < 0.001).

## 5. Conclusions

Overall, our results establish an accurate and integrative framework for the rapid evaluation of CRISPR reagents that can be readily adapted to other crop species. In particular, given the phylogenetic proximity and shared transformation challenges, this approach may be especially transferable to members of the Asteraceae family, where efficient genome editing platforms remain underdeveloped. More broadly, this workflow provides a valuable foundation for implementing similar strategies to accelerate the development and optimization of gene editing systems across diverse crops.

At the same time, it is important to acknowledge that as with other CRISPR validation systems, hairy root-based approaches may present certain limitations, including potential discrepancies between editing outcomes observed in hairy roots and those in whole plants. Further studies will be necessary to systematically assess these differences and to determine the extent to which hairy roots can serve as a reliable predictive platform for stable genome editing outcomes.

## Figures and Tables

**Table 1:** Comparison between predicted sgRNA efficiency and in planta genome editing in transformed hairy roots.

		Computational Prediction ^a^					In Planta ^b^			
Nuclease	Guide	Fusi/Doench/Azimuth	Crispr Scan	Najm 2018	deepCpf1	Out-of-Frame	Edited HR/Total HR	Indel %	KO Score	KO Score Normalized
SpCas9	Sp-sg73	57	60	-	-	75	85	55.4	54.7	98.7
	Sp-sg74	66	56	-	-	68	62	55.5	51.6	93.0
SaCas9	Sa-sg27	-	-	91	-	66	90	64.3	58.1	90.4
LbCas12a	cr56	-	-	-	30.81		100	65.6		
	cr61	-	-	-	78.78		100	73		
	cr101	-	-	-	64.95		89	57.1		

^a^ Computational prediction represents in silico results of the sgRNAs’ predicted efficiency scores generated from CRISPOR; scores are rated from 0 to 100, and higher scores indicate higher efficiency. Fusi/Doench/Azimuth [66] and CrisprScan [14] algorithms predict the activity of SpCas9 sgRNAs; Najm 2018 [20] is a modified version of the Doench 2016 [66] score for *S. aureus* Cas9; DeepCpf1 [19] predicts the activity of Cas12a crRNAs; Out-of-frame [67] predicts the frequency of out-of-frame caused by indels. ^b^ On the right of the table is shown ‘in planta’ editing performance. Edited HR/total HR indicates the proportion of hairy roots harboring at least one edited allele; indel% and KO score derived from ICE analysis indicate the total percentage of indel alleles and the total percentage of knockout alleles in hairy roots, respectively; the normalized KO score represents the percentage of KO mutation present in indels.

## Data Availability

The original contributions presented in this study are included in the article/Supplementary Material. Further inquiries can be directed to the corresponding authors.

## Supplementary Materials

The following supporting information can be downloaded at https://www.mdpi.com/article/10.3390/plants15081161/s1. Figure S1: PCR genotyping of hairy roots; Table S1: ICE data; Table S2: Primers for level 0 modules; Table S3: Primers for level 1 modules; Table S4: Primers for sgRNAs SpCas9 and SaCas9; Table S5: Primers for crRNA Cas12a; Table S6: Constructs; Table S7: Primers for hairy root genotyping; Table S8: In silico off-target editing analysis; Table S9: Contingency table of explants forming hairy roots for each combination of cultivar and *A. rhizogenes* strain and statistical analyses; Table S10: Contingency table of hairy roots transformed with 2×p35S::GFP:NLS::T35S and pPcUbi::GFP:NLS::T35S showing the number of roots; Table S11: Indel frequency generated by six different constructs in lettuce hairy roots and statistical analysis; Table S12: Contingency table showing editing frequencies and the distribution of genotypic classes in hairy roots obtained using different combinations of guide RNAs and nucleases, as determined by ICE analysis. File S1: Cloning procedure.
