# Supplementary material for: A Rapid Hairy Root-Based Platform for CRISPR/Cas Optimization and Guide RNA Validation in Lettuce
Source: Plants (Basel). 2026 Apr 9;15(8):1161. doi: 10.3390/plants15081161 (PMC13119113; doi:10.3390/plants15081161)
Supplement: Supplementary file 1 [file plants-15-01161-s001.zip › Supplementary File S1.pdf]

A rapid hairy root–based platform for CRISPR/Cas optimization and guide RNA validation in lettuce

Alberico Di Pinto*, Valentina Forte*, Chiara D'Attilia, Marco Possenti, Barbara Felici, Floriana Augelletti, Giovanna Sessa, Monica Carabelli, Giorgio Morelli, Giovanna Frugis and Fabio D'Orso

Supplementary File S1

Cloning procedure

BpiI- or BsaI-mediated DIG–LIG reactions were carried out in a final volume of 7.5 µL, containing 50 ng of acceptor vector, inserts at a 3:1 molar ratio relative to the acceptor vector, 5 U of BpiI or 10 U of BsaI depending on the specific reaction, 100 U of T4 DNA ligase, 1× T4 DNA ligase buffer, and 1× BSA. Esp3I-mediated DIG-LIG reactions were performed in a final volume of 10 µL containing 200 ng of acceptor vector, 2 µM oligonucleotide pairs denatured at 95°C for 5 min and reannealed at room temperature, 10 U of Esp3I, 200 U of T4 Ligase, 1X T4 Ligase buffer, 1X BSA. About level 0 modules, AtU6-26 promoter, PcUbi promoter, Pea3A terminator and GFP coding sequence fused with Nuclear Localization Signal peptide (NLS) were PCR amplified and cloned into pUAP1 universal level 0 acceptor by BpiI-mediated DIG-LIG reaction. Templates and primers used for amplifications are reported in the Table S2. Level 1 modules for the 35S::GFP and PcUbi::GFP expression cassettes were assembled by BsaI-mediated DIG–LIG reactions, combining the respective Level 0 modules into the Level 1 acceptor vector pICH47742, as indicated in Table S6. Level 1 cloning of nuclease expression cassettes was performed by amplifying the SpCas9, SaCas9, and LbCas12a coding sequences, flanked by the PcUbi promoter and the Pea3A terminator, from the plasmids pDe_CAS9, pDe-Sa-CAS9, and pDe_LbCpf1_PPT, respectively (Puchta's lab), using the primers listed in Table S3. The resulting PCR products were cloned into the Level 1 acceptor vector pICH47742 by DIG–LIG reactions (Table S6). sgRNAs for SpCas9 or SaCas9 were assembled by PCR by using the pICSL70001 or pEn-Sa-Chimera plasmids as template for SpsgRNA or SasgRNA scaffolds, respectively (Table S4). The resulting amplicons were assembled into the level 1 acceptor pICH47751 together with pU6 promoter provided by pICSL90001 (for SpsgRNAs only) or FD119-AtpU6-26_L0 (for both SpsgRNAs and SasgRNAs) by BsaI-mediated DIG-LIG reaction (Table S6). Level 1 module for preassembled AtpU6-26-ribozyme-LbcrRNAscaffold-lacZ-ribozyme (FD118) was generated by fusing three parts: (i) AtpU6-26-ribozyme-LbcrRNAscaffold, amplified from pEn_RZ_Lb_Chimera (Puchta's lab), (ii) lacZ expression cassette amplified from pICH47732 and (iii) ribozyme amplified from pEn_RZ_Lb_Chimera, using the primers listed in Table S3. These parts were assembled into pICH47751 level 1 acceptor by BsaI-mediated DIG-LIG reaction and the resulting construct served as level 1 acceptor for crRNA spacer insertion using oligonucleotides with overhangs complementary with those generated by cutting FD118 plasmid with Esp3I restriction enzyme. For each LbcrRNA, LacZ was removed from FD118 and replaced with the respective spacers by Esp3I-mediated DIG-LIG reaction to make a complete LbcrRNA expression cassette. Sequences of oligonucleotide pairs which constitute crRNA spacers are reported in the table S5. Level 2 assemblies for final expression constructs were performed by BpiI-mediated DIG-LIG reactions (Table S6).
