# Supplementary material for: A Rapid Hairy Root-Based Platform for CRISPR/Cas Optimization and Guide RNA Validation in Lettuce
Source: Plants (Basel). 2026 Apr 9;15(8):1161. doi: 10.3390/plants15081161 (PMC13119113; doi:10.3390/plants15081161)
Supplement: Supplementary file 1 [file plants-15-01161-s001.zip › Supplementary_Figure_S1.pdf]

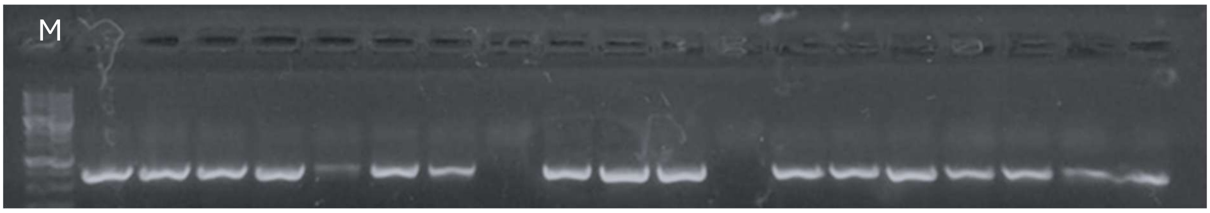

Supplementary Figure S1. PCR genotyping of hairy roots. Genomic DNA from individual hairy roots was screened via PCR using specific primers to amplify a 786 bp fragment of the *nptII* kanamycin resistance gene (NPTII-FW 5’-GAACAAGATGGATTGCACGC-3’; NPTII-REV 5’- GAAGAACTCGTCAAGAAGGC-3’). Genomic DNA was amplified directly from hairy roots using a commercial kit (Phire Plant Direct PCR Kit; Thermo Scientific, USA). The image shows an example of PCR genotyping of hairy roots from the ‘Osiride’ cultivar. M: molecular weight marker (1 Kb Gene Ruler, Thermo Scientific, USA).
